# Room Temperature Light Emission from Superatom-like Ge–Core/Si–Shell Quantum Dots

**DOI:** 10.3390/nano13091475

**Published:** 2023-04-26

**Authors:** Katsunori Makihara, Yuji Yamamoto, Yuki Imai, Noriyuki Taoka, Markus Andreas Schubert, Bernd Tillack, Seiichi Miyazaki

**Affiliations:** 1 Graduate School of Engineering, Nagoya University, Furo–cho, Chikusa–ku, Nagoya 464-8603, Japan; 2 IHP–Leibniz-Institut für Innovative Mikroelektronik, Im Technologiepark 25, 15236 Frankfurt, Germany; yamamoto@ihp-microelectronics.com (Y.Y.);; 3 Technische Universität Berlin, HFT4, Einsteinufer 25, 10587 Berlin, Germany

**Keywords:** Si quantum dots, core/shell structure, CVD

## Abstract

We have demonstrated the high–density formation of super–atom–like Si quantum dots with Ge–core on ultrathin SiO_2_ with control of high–selective chemical–vapor deposition and applied them to an active layer of light–emitting diodes (LEDs). Through luminescence measurements, we have reported characteristics carrier confinement and recombination properties in the Ge–core, reflecting the type II energy band discontinuity between the Si–clad and Ge–core. Additionally, under forward bias conditions over a threshold bias for LEDs, electroluminescence becomes observable at room temperature in the near–infrared region and is attributed to radiative recombination between quantized states in the Ge–core with a deep potential well for holes caused by electron/hole simultaneous injection from the gate and substrate, respectively. The results will lead to the development of Si–based light–emitting devices that are highly compatible with Si–ultra–large–scale integration processing, which has been believed to have extreme difficulty in realizing silicon photonics.

## 1. Introduction

Monolithic integration of Si/Ge–based optoelectronic devices into Si–ULSI circuits is of great interest to further extend the functionality of complementary metal–oxide–semiconductor (CMOS) technologies according to a “more than Moore” approach [1,2,3,4,5,6,7,8,9,10,11,12,13,14,15,16]. Thus, light emission from Si/Ge–based quantum dots (QDs) has attracted much attention as an active element of optical applications because it has the advantages of photonic signal processing capabilities and combines them with electronic logic control and data storage. In recent years, intensive research has been dedicated to the growth and optoelectronic characterization of Si and/or Ge nanostructures using various fabrication techniques such as molecular beam epitaxy, chemical vapor deposition (CVD), and magnetron sputtering deposition [17,18,19,20,21]. However, the development of a growth technology for QDs with high areal density, uniform shape, and narrow size distribution for large–scale production is a crucial factor in realizing stable light emission from the QDs. In addition, to integrate optoelectronic devices into the CMOS technology, remarkable improvements in light emission efficiency and its stability for practical use are major technological challenges because of the difficulty in achieving a good balance between charge injection and confinement in the QDs. To satisfy both strong confinement and smooth injections of carriers, Ge–QDs and their self–aligned stack structures embedded in Si have so far been fabricated by controlling strain–induced self–assembling in the early stages of Ge heteroepitaxy on Si. So, we have focused on superatom–like Ge–core/Si–shell QDs (Si–QDs with Ge–core) formed on ultrathin SiO_2_ because of their potential to realize a good balance between charge injection and confinement in the QDs, which can improve light emission efficiency and stability. Additionally, defect control at interfaces between Si and SiO_2_ is established. In our previous works, we have studied the highly selective growth of Ge on pre–grown Si–QDs and the subsequent coverage with a Si–cap that enables us to superatom–like Ge–core/Si–shell QDs and characterized their unique electron/hole storage properties, reflecting the type II energy band discontinuity between Si–QDs and Ge core [22,23,24,25,26]. More recently, we also demonstrated photoluminescence (PL) from the Si–QDs with Ge–core and discussed the origin of their PL properties, with PL having less dependence on temperature change. Therefore, we concluded that hole confinement in the Ge core plays an important role in radiative recombination in Si/Ge QDs. In addition, we also reported electroluminescence (EL) from the light–emitting diode structure with the 3–fold stacked Si–QDs with Ge core by application of continuous square–wave pulsed bias under cold light illumination, where EL signal was observed from the backside through the c–Si substrate caused by alternate electron/hole injection from the p–Si(100). In this work, we developed fabrication processes of the Si–QDs with Ge–core structure by means of a commercial reduced pressure (RP) CVD system [27,28,29,30] using SiH_4_ and GeH_4_ gases with H_2_ carriers, which has been in practical use for the mass production of bipolar CMOS, for the fabrication of the Si–QDs with Ge–core on ultrathin SiO_2_ layers, and evaluated their PL and EL characteristics without light illumination at room temperature.

## 2. Materials and Methods

The fabrication of Si–QDs with Ge–core was carried out using an RPCVD system. After a standard RCA cleaning step, ~7–nm–thick SiO_2_ was grown on a p–Si(100) wafer by H_2_/O_2_ oxidation at 650ºC. Furthermore, diluted HF dip is performed. At this point, residual SiO_2_ thickness is ~2.0 nm. After that, the SiO_2_/Si(100) wafer was loaded into the RPCVD reactor and baked at 850ºC in RP–H_2_ to control OH bond the SiO_2_ surface. Subsequently, the wafer was cooled down to 650ºC in RP–H_2_. After temperature stabilization, Si–QDs were deposited using a H_2_–SiH_4_ mixture gas by controlling the early stages of Si nucleation. Directly after that the wafer was cooled down to 550 °C in the RP–H_2_ environment and Ge was selectively grown on the pre–grown Si–QDs by H_2_–GeH_4_. Afterwards, the wafer was heated again up to 650 °C in a H_2_ environment and Si cap was selectively deposited on the Ge on the Si–QDs by H_2_–SiH_4_ gas system. In order to maintain selectivity, lower SiH_4_ partial pressure is used for the Si cap growth.

Areal density and average height of the Si–QD with Ge–core were evaluated by atomic force microscopy (AFM), where average dot heights were determined by log–normal functions. The dot height of the Si and Ge in the Si–QDs with a Ge–core was also characterized by cross–section transmission electron microscopy (TEM) and energy–dispersive X–ray spectroscopy (EDX) mapping. PL measurements were carried out by using a 976–nm light as an excitation source with an input power of 0.33 W/cm^2^.

For the fabrication of the LED structure, ~200–nm–thick amorphous Si was deposited on the Si–QDs with Ge–core by electron beam evaporation at room temperature after the chemical oxidation of the dots surface. Then, phosphorus atom implantation at 45 keV was conducted. Subsequently, the sample was annealed at 300 °C. Then, the amorphous Si layer was etched by Cl_2_ plasma to isolate each device. Finally, ring–patterned Al–top electrodes with an aperture of ~78.5 mm^2^ and backside electrodes were fabricated by thermal evaporation. For the PL and EL measurements, thermoelectrically cooled InGaAs photodiodes were used as detectors in this work.

## 3. Results and Discussion

**Figure 1:**
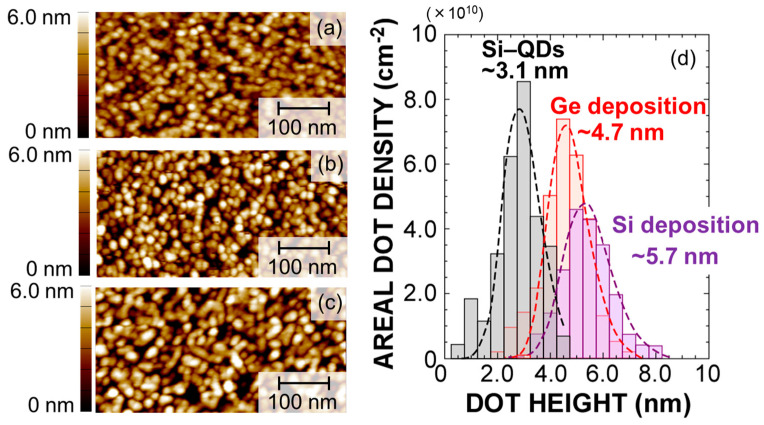
Typical AFM images of (**a**) pre–grown Si–QDs, (**b**) after Ge deposition, (**c**) after Si–cap formation, and (**d**) dot height distribution of the samples shown in (**a**–**c**). The corresponding curves denote log–normal functions well–fitted to the measured size distribution.

**Figure 2:**
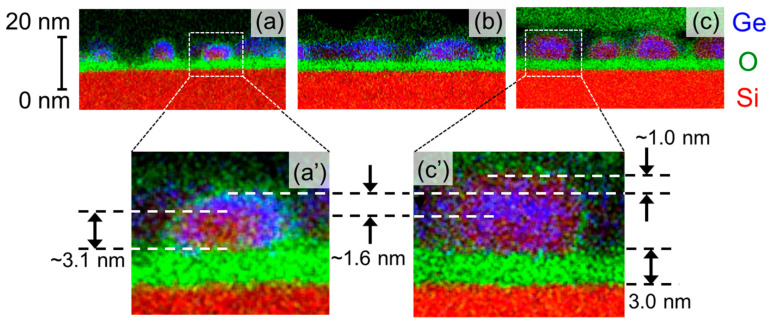
Cross–sectional EDX mapping images of (**a**) Ge deposition on the pre–grown Si–QDs, (**b**) after annealing at 650 °C, and (**c**) Si–cap formation at 650 °C. Images (**a’**) and (**c’**) are enlarged view corresponding to (**a**) and (**c**), respectively.

**Figure 3:**
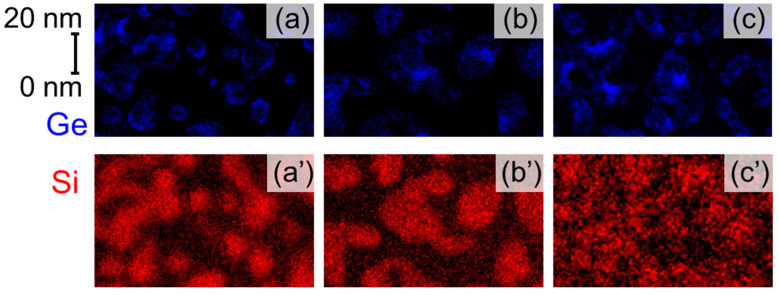
Room temperature PL spectra of the Si–QDs with Ge–core and their deconvoluted spectra evaluated from the spectral analysis using the Gaussian curve fitting method. In–plane EDX mapping images of (**a**) and (**a’**) Ge deposition on the pre–grown Si–QDs, (**b**) and (**b’**) after annealing at 650 °C, and (**c**) and (**c’**) Si–cap formation at 650 °C. Blue and red colors are corresponding to Ge and Si, respectively.

The formation of high–density Si–QDs with Ge–core on the ultrathin SiO_2_ surface was confirmed by AFM and EDX mapping image measurements, as shown in Figure 1 and Figure 2. The AFM topographic image taken after the RPCVD using a H_2_–SiH_4_ mixture gas at 650 °C confirms that hemispherical Si–QDs with an areal density as high as ~1.0 × 10^11^ cm^−2^ were self–assembled on the SiO_2_ surface. Additionally, the AFM images taken after each deposition step confirm that the areal dot density remains unchanged after the Ge deposition and subsequent Si deposition using the high selectivity process condition. These results indicate that the Ge deposition and the Si–cap formation occur very selectively on each of the isolated dots, and no new dots nucleate on the SiO_2_ layer during the Ge deposition and Si–cap formation. From dot height distributions evaluated from cross–sectional topographic images, we also confirm that the dot height is increased by ~1.6 nm and by ~1.0 nm after the Ge deposition and subsequent Si–cap formation, respectively. In our previous report on the formation of the Si–QDs with Ge–core using LPCVD [26], the full–width at half–maximum value of the size distribution after Si–cap formation is ~7 nm, whereas it is ~3 nm for RPCVD, indicating that dots of extremely uniform size can be formed. Highly selective deposition of Ge and subsequent Si on the dots was also verified from the cross–section TEM–EDX analysis, as indicated in Figure 2. After the H_2_–GeH_4_–RPCVD, an EDX mapping image shows that Ge was deposited on the pre–grown Si–QDs conformally, although there is no deposition on the SiO_2_ surface in–between the pre–grown Si–QDs. After the subsequent H_2_–SiH_4_–RPCVD, Ge–core/Si–shell structure was clearly detected. In addition, the heights of the pre–grown Si–QD, Ge–core, and Si–cap are very consistent with the values estimated from the size distribution shown in Figure 1d. It is interesting to note that the EDX mapping image of the dots after the Si–cap deposition shows that Ge has a rather spherical shape in contrast to the as–deposited Ge selectively on pre–grown hemispherical–shaped Si–QDs. To get an insight into the change in the Ge–core shape, we also performed plane–view TEM–EDX analysis, as shown in Figure 3. After the Ge deposition, the color contrast of the Ge in the peripheral region of the dots is somewhat deeper than that in the center of the dots, which indicates that Ge was deposited conformally on the pre–grown Si–QDs because color contrast depends on the thickness. Contrary to this, after annealing at 650 °C and/or Si–cap deposition at 650 °C, it turned out to have a dark color in the central part. This result can be attributable to Ge reflow onto the pre–grown Si–QDs due to relaxation of surface energy or high structural strain at the Ge/Si interface. Consequently, a spherical–shaped Ge–core with an abrupt Ge/Si interface was formed. In fact, Raman scattering spectra for the dots indicate that compositional mixing hardly occurs at Si/Ge interfaces during the sample preparation, as verified by the relative intensity of the Si–Ge phonon mode with respect to the Si–Si and Ge–Ge phonon modes (not shown).

**Figure 4:**
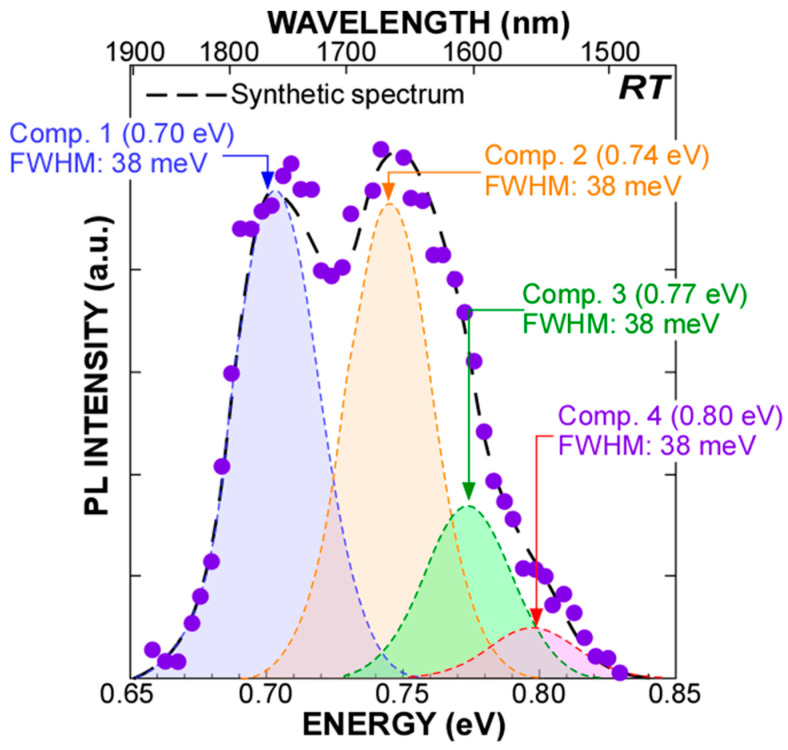
Room temperature PL spectra of the Si–QDs with Ge–core and their deconvoluted spectra evaluated from the spectral analysis using the Gaussian curve fitting method.

**Figure 5:**
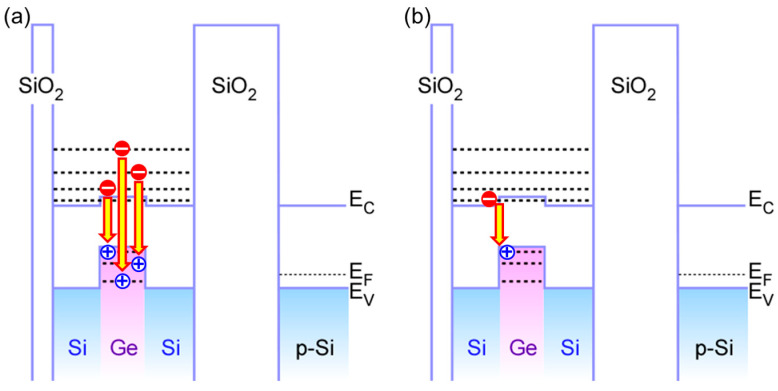
Energy band diagram of Si–QDs with Ge–core, illustrating radiative transition corresponding to (**a**) PL components 2–4, and (**b**) component 1 as shown in Figure 4.

Under 976–nm light excitation at an input power of 0.33 W/cm^2^ of the Si–QDs with Ge–core using a semiconductor laser, a stable PL signal consisting of four Gaussian components in the energy region from 0.66 to 0.83 eV was detected at room temperature, as shown in Figure 4. As verified from the dot size dependence of PL peak energy and the temperature dependence of PL properties discussed in [25], these components are attributable to radiative recombination through quantized states in QDs. Based on our previous discussion, Comp. 2 is attributable to the radiative recombination between the conduction and valence bands of the Ge–core through the first quantized states, as shown in Figure 5a. Therefore, providing that the selection rule in quantum mechanics is valid, the higher energy components, Comp. 3 and 4, can be explained by the radiative transition between the higher order quantized states in the conduction and balance bands of Ge core. Considering type II energy band diagram of the Ge–core/Si–shell structure, component 1 might be attributable to radiative recombination between the quantized state of electron in the Si clad and the quantized state of hole in the Ge core because the Si clad acts as a shallow potential well for electron in which electron wave function can penetrate the Ge core, as indicated in Figure 5b. It should be noted that the full width at half maximum (FWHM) values of these components are ~38 meV for all components, which can be explained by a size variation of the Ge–core. However, compared with our previous report, where PL signal in the range from 0.65 to 0.88eV was observed from the Si–QDs with Ge–core formed by a low–pressure CVD, the observed PL spectra in this work are narrower due to the formation of extremely uniform–sized dots.

**Figure 6:**
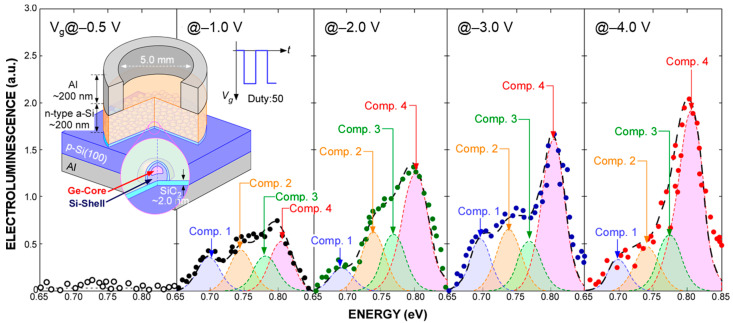
Room temperature EL spectra taken at different applied biases from LEDs having Si–QDs with Ge–core and their deconvoluted spectra evaluated from the spectral analysis using the Gaussian curve fitting method. A schematic illustration of the LED is also shown in the inset.

**Figure 7:**
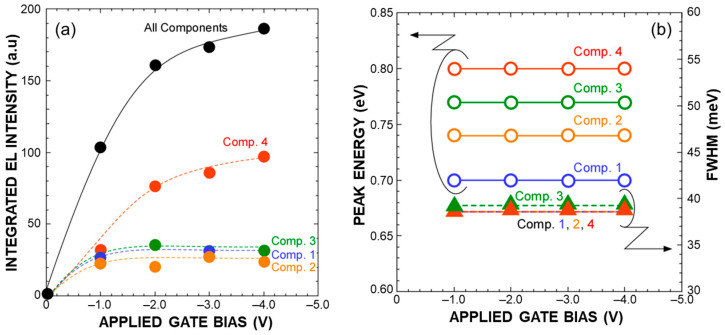
Integrated EL intensities (**a**) and EL peak energy and full width at half maximum of each EL component (**b**) evaluated from the spectral analysis using a Gaussian curve fitting method as a function of applied bias.

The I–V characteristics of light–emitting diodes (LEDs) on p–Si(100), as schematically illustrated in the inset of Figure 6, show a clear rectification property reflecting the work function difference between the n–type amorphous Si electrode and p–Si(100) substrate (not shown). With the application of continuous square–wave pulsed bias in the negative half cycle with peak–to–peak amplitude over 1.0 V to the top electrodes, EL signals having similar components to PL signals became observable from the topside of the LED structure through the a–Si layer even at room temperature, as shown in Figure 5. Notice that the observed EL spectra consist of four Gaussian components. It should be noted that their peak energies and FWHM values are almost the same as those as the corresponding components evaluated from the PL signal shown in Figure 4 were detected at room temperature. This suggests that the recombination mechanism for the EL is the same as that of the PL. In addition, a significant change in each of the peak positions was confirmed with an increase in applied bias. No EL signals were detected in the positive half cycle bias condition. Therefore, the EL can be explained by radiative recombination of electrons and holes caused by electron injection from the P–doped a–Si and hole injection from the p–Si(100). With an increase in the bias amplitude, the EL intensity increased with almost no change in the peak energy of each component, and then higher emission energy components became dominant, as shown in Figure 7. It should be noted that, by applying a square–wave bias of −4 to 0 V, the EL signal peaked at ~0.8 eV, which is the same energy as that of component 4 evaluated from the PL spectrum is dominant, implying that electrons and holes were injected into higher–order quantized states. From the negative–bias amplitude dependence of the EL intensity, we have found that radiative recombinations between higher–order quantized states become a major factor for EL at −4 V as a result of which single–peak emission can be realized. Results obtained in this research will lead to the realization of Si–based optoelectronic devices by introducing a Ge–core into the Si–QDs (pseudo–super atom structure), which has the advantage of stimulating radiation due to a narrow emission wavelength spectrum in a low–voltage application.

## 4. Conclusions

In summary, high–density Si–QDs with Ge–core show PL in the near–infrared region at room temperature, which indicates that the hole confinement in Ge–core plays an important role in radiative recombination into the Ge–core. We have also demonstrated stable EL in the near–infrared region from light–emitting devices having Si–QDs with a Ge–core caused by electron injection from the top electrodes simultaneously with hole injection from the substrate under continuous square–wave pulsed bias in negative half–cycle applications. From a technological point of view, it is quite important that such a stable EL caused by electron/hole simultaneous injection from the gate and substrate, respectively, at room temperature was realized by using Si–QDs with Ge core, which is compatible with Si–ULSI processing.

## Data Availability

Not applicable.
